# Post-Processing Time Dependence of Shrinkage and Mechanical Properties of Injection-Molded Polypropylene

**DOI:** 10.3390/ma14010022

**Published:** 2020-12-23

**Authors:** Artur Kościuszko, Dawid Marciniak, Dariusz Sykutera

**Affiliations:** Department of Manufacturing Techniques, UTP University of Science and Technology, Kaliskiego 7, 85-796 Bydgoszcz, Poland; artkos@utp.edu.pl (A.K.); sykutera@utp.edu.pl (D.S.)

**Keywords:** injection molding, polypropylene, post-molding shrinkage, 3D scanning, mechanical properties

## Abstract

Dimensions of the injection-molded semi-crystalline materials (polymeric products) decrease with the time that elapses from their formation. The post-molding shrinkage is an effect of secondary crystallization; the increase in the degree of polymer crystallinity leads to an increase in stiffness and decrease in impact strength of the polymer material. The aim of this study was to assess the changes in the values of post-molding shrinkage of polypropylene produced by injection molding at two different temperatures of the mold (20 °C and 80 °C), and conditioned for 504 h at 23 °C. Subsequently, the samples were annealed for 24 h at 140 °C in order to conduct their accelerated aging. The results of shrinkage tests were related to the changes of mechanical properties that accompany the secondary crystallization. The degree of crystallinity of the conditioned samples was determined by means of density measurements and differential scanning calorimetry. It was found that the changes in the length of the moldings that took place after removal from the injection mold were accompanied by an increase of 20% in the modulus of elasticity, regardless of the conditions under which the samples were made. The differences in the shrinkage and mechanical properties of the samples resulting from mold temperature, as determined by tensile test, were removed by annealing. However, the samples made at two different injection mold temperature values still significantly differed in impact strength, the values of which were clearly higher for the annealed samples compared to the results determined for the samples immediately after the injection molding.

## 1. Introduction

The injection process is commonly used to produce polymer products, even with complex shapes. The injection molded parts are expected to have required dimensions within the accepted tolerance range [1]. The moldings should be free from sink marks, voids and warpage [2,3,4] after the end of the processing cycle. Moreover, the dimensions and shape of the moldings should be maintained throughout their lifetime. The main problem of precise mapping of the dimensions and shape of the injection mold cavity is processing induced shrinkage, which is a measure of the volume and shape changes due to the cooling conditions by injection molding. Processing shrinkage is defined as the difference (expressed as a percentage) between the dimensions of the mold cavity and the dimension of the molded part (at 23 ± 2 °C), related to the cavity dimension. The test of the shrinkage of the moldings is usually performed 16–24 h after the end of processing cycle (representing the primary shrinkage). Nevertheless, after this time, the dimensions of the injection moldings of several polymeric materials are not fully stabilized. Depending on the storage and/or conditions of use, the dimensional changes of moldings over time may continue up to 1000 h from their production, as the secondary shrinkage [5]. This applies in particular to semi-crystalline materials whose glass transition temperature is lower than the service temperature. 

Shrinkage is caused by a gradual decrease in the volume of the polymer material during the molded state, as well as after its removal from the mold cavity. The change in the volume of the material is a consequence of thermal expansion. Moreover, the partially crystalline polymers undergo a transformation from an amorphous liquid phase to a partially crystalline solid phase. To describe the phenomena occurring during contraction, graphs of the relationship between pressure (*p*), volume (*v*) and temperature (*T*) are used [6,7]. The content of individual phases in finished products significantly affects their physical properties [8]. An effect of the development of the well-organized structure of macromolecules during crystallization is an increase in the density of the material. Ducić [9] showed that the density of fully crystalline polypropylene (PP) is 0.936 g/cm^3^ and is about 7% higher compared to amorphous PP (0.869 g/cm^3^). A modification of the crystal structure of polypropylene, and thus of mechanical properties, can be achieved by heterogeneous nucleation [10,11]. The development of various crystalline forms, due to the polymorphic character of isotactic polypropylene (iPP), was observed and widely described [12,13,14,15]. A number of studies have been conducted to reduce the shrinkage value of injection moldings [16,17,18]. In the case of thick-walled products, the porous process of polymeric materials is often used. In practice, two porous methods are used: chemical [19,20] and physical (microcellular injection molding) [21,22]. However, the use of this type of modification may reduce the tensile strength and impact strength [23,24] of the polymeric material.

Studies involving the assessment of the influence of the post-processing time on the properties of PP have already been conducted by many researchers [25,26,27]. The authors usually considered long-term conditioning at temperature between 21–23 °C as a type of aging test. Gahleitner [28] considered changes in impact strength and flexural modulus for PP moldings conditioned for less than 10,000 h at 23 °C. Yue [29] conducted the aging process for 6000 h (250 days), testing the stress at yield point and determining the degree of crystallinity of the samples by using the differential scanning calorimetry (DSC) measurements. About a 20% increase of stress at yield was observed for compression molded parts. In contrary to Yue’s work, the results of the investigations published by Sližová [30] seem surprising. The author indicates that after 30 years of storage under changing conditions, the stress at yield point of PP moldings has not changed.

The research consisting of the assessment of the influence of the conditioning time on the structure and mechanical properties of polypropylene was usually carried out on films [28], samples cut from sheets or bars or samples obtained by compression molding [9,29,31]. Moreover, the PP structure was often stabilized by annealing prior to the main aging tests. There are a few research studies in this area that deal with the subject of changes occurring in structure and properties of the samples made by injection molding during their conditioning. The samples prepared by the injection method at the variable temperature of the injection mold are used primarily in research aimed at assessing the influence of this process parameters on the structure and mechanical properties of molded parts [32,33]. PP samples produced at a high temperature of the injection mold are usually characterized by a higher degree of crystallinity and stress at the yield point. However, no publication has been found that describes the relationship between the samples’ preparation conditions with the injection molding method, the conditioning time and shrinkage, structure and mechanical properties, using the example of PP molded parts with the same weight.

The main aim of this research was to assess the mechanical properties of PP thick-walled molded parts in relation to changes of their shrinkage during post-molding time. The tests were carried out for moldings having the same mass, formed at two different temperature values of the injection mold. Apart from traditional measuring tools, the 3D optical scanning method was used to evaluate the post-molding shrinkage. Moreover, the changes in dimensions and mechanical properties of the test samples were compared with changes in the structure. The results of the carried out research supplement the knowledge on the relationship between the injection moldings parameters and the structure, as well as mechanical properties of conditioned thick-walled PP molded parts.

## 2. Materials and Methods 

### 2.1. Material

Commercial grade of Moplen HP 500N, an isotactic polypropylene by Basell Orlen Polyolefins (Płock, Poland), was used in our research. This material is proposed for processing by injection molding. The mass flow rate (MFR) of Moplen HP 500N was 12 g/10 min (230 °C, 2.16 kg); the modulus of elasticity (*E*) and tensile strength (*Rm*), as declared by the manufacturer, were 1550 MPa and 35 MPa, respectively. The polymer was not modified by nucleation.

### 2.2. Samples Preparation

The test specimens were made by injection molding using the Engel (Schwertberg, Austria) e-victory 110 hybrid injection molding machine, with a clamping force of 1100 kN. We have used a four-cavity injection mold [34], allowing for the production of universal A-type test samples with their dimensions corresponding to ISO 3167 standard. The length at the mold cavity (*L_F_*) was 168 mm. The thickness of the obtained samples was about 4 mm. The samples were produced at two different injection mold temperature values (*T_F_*), equal to 20 °C (designation PP20) and 80 °C (designation PP80), respectively. A mold temperature of about 20 °C is commonly used in industrial practice to produce PP products, while about 80 °C is the upper limit of the mold temperature that can be used for this type of material. The injection mold was thermostated using the *HB-160Z1* device produced by HB-Therm (St. Gallen, Switzerland). The temperature of individual zones of the plasticizing system was 230 °C (nozzle), 230 °C, 220 °C, 200 °C and 40 °C at the feed zone. The injection rate was 50 cm^3^/s and the holding pressure was 19 MPa. The holding time was set in such a way to assure a similar weight of individual test samples (8.60 ± 0.02 g), regardless of temperature value of the injection mold. For the mold temperature of 20 °C, the holding time was 21 s, and the cooling time was 46 s, while for the *T_F_* equaling 80 °C, these values were 45 s and 70 s, respectively. As mentioned above, the differentiation of the holding time was important in obtaining test samples of similar mass. It should be emphasized that in the injection molding, increasing the temperature of the mold, while maintaining constant values of other process parameters, primarily the same duration of the holding phase, results in obtaining moldings of a lower mass. This is due to the phenomenon of PP thermal expansion. By increasing the temperature of the injection mold, it is possible to extend the duration of the holding phase, which is practiced in the industry to increase the dimensional accuracy of molded parts. The result is an increase in mass compared to the mass of molded parts obtained at a lower mold temperature. The samples were conditioned for the period of 504 h at 23 °C and air humidity RH = 50%. After this time, the samples were heated for 24 h at 140 °C (samples signature—504 h A).

### 2.3. Measurements of Moldings Shrinkage

The values of linear processing shrinkage (*S*) were calculated on the basis of the samples length (*L_T_*) with the use of the following formula:

(1) $$S=\;\frac{L_{F-\;}L_{T}}{L_{F}}\;\times 100\%.$$

The measurements were carried out by means of MarCal 16ER caliper by Mahr (Göttingen, Germany) after 1 h, 24 h, 48 h, 72 h, 168 h (7 days), and finally after 504 h (21 days) from the formation of the moldings, at 23 °C always for 10 samples from each measurement series. Moreover, the geometry of the samples was measured after 24 h of annealing at 140 °C, 1 h after the end of annealing. The study of the shrinkage of specimens produced at the mold temperature of 20 °C and 80 °C, characterized by a similar weight, was aimed to assess the impact of processing conditions and the conditioning time on the shrinkage value of PP moldings.

**Figure 1:**
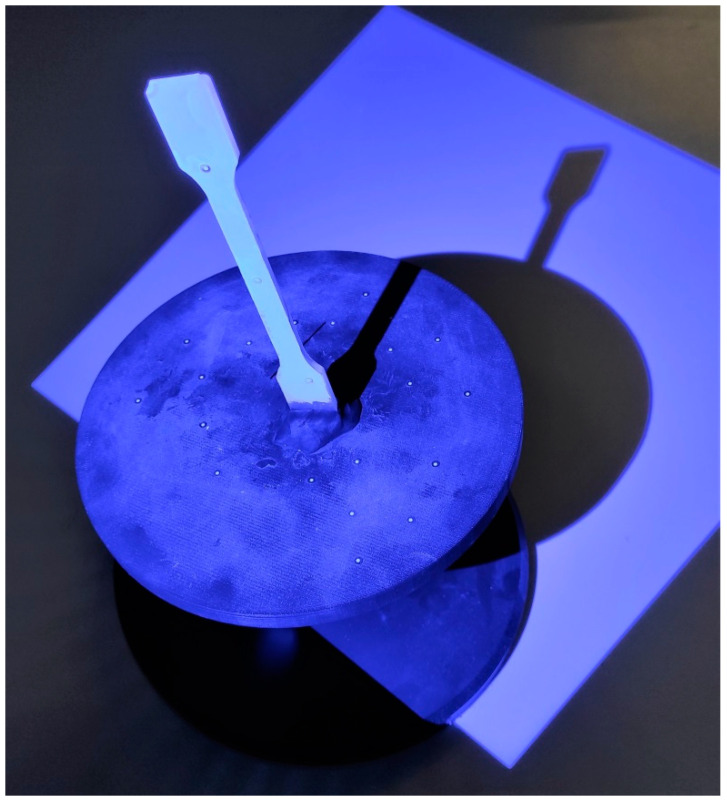
Polypropylene sample prepared for the 3D scanning.

Moreover, the shrinkage of the PP samples was investigated using the 3D Atos Triple Scan optical scanner by GOM (Braunschweicg, Germany) equipped with cameras with a resolution of 5 MPx. The preparation of test samples for measurement consisted of marking the surface with reference points with a diameter of 1.5 mm, and in matting, the surface with a powder applied with an aerosol (Figure 1). The geometry measurements were carried out for samples conditioned for 1 h, 504 h, as well as after 24 h of annealing. Using the GOM Inspect Siute 2020 software, a comparison between the surface geometry of PP20 sample (the reference sample) and PP80 sample after 1 h of conditioning, was carried out in order to determine the deviations from the reference surface (Δ*h*). A detailed analysis of the differences in dimensions was performed for 5 points located along the symmetry axis of the 3 samples, i.e., in the measurement section of the test samples. Moreover, the comparison of the samples geometry after 504 h of conditioning and after annealing was made. The PP20 and PP80 samples after 1 h of conditioning were the reference samples in this case.

### 2.4. Measurements of Density

The density of polypropylene samples was determined by an immersion method. The measurements were carried out with the use of AD 50 balance (Axis, Grańsk, Poland), equipped with a set allowing to determine the mass of the samples both, in the air and in the immersion liquid, which was methyl alcohol with a density of 0.792 g/cm^3^. The tests were performed at an ambient temperature of 23 °C for 10 samples after 1 h and 504 h from removing the samples from the injection mold and after annealing of the samples. The density for single samples was evaluated based on the following formula:

(2) $$\rho =\frac{m\times \rho_{L}}{m-m_{L}}$$

where the symbols *m* and *m_L_* denote the mass of the sample determined in the air and in the immersion liquid, where the symbol *ρ_L_* denotes the density of the immersion liquid. The crystallinity of the conditioned PP samples was determined using the density, by means of following formula:

(3) $$X_{\rho }=\frac{\rho_{C}}{\rho }\times \left(\frac{\rho -\rho_{A}}{\rho_{C}-\rho_{A}}\right)\times 100\%$$

where *ρ* denotes the density of the tested material and the symbols *ρ_C_* and *ρ_A_* denotes the density of the 100% crystalline (0.936 g/cm^3^) and amorphous (0.856 g/cm^3^) phases of PP [9].

### 2.5. Measurements of the Melting Behavior

The tests of the thermal properties of the PP samples were carried out by differential scanning calorimetry (DSC) using the DSC 214 Polyma apparatus by Netzsch (Selb, Germany). Samples weighing 8–10 mg were heated to 220 °C in a nitrogen atmosphere. After a two-minute exposure at the set temperature, they were cooled to 25 °C. The heating and cooling rates were 10 °C/min. The tests were carried out for three PP samples after 1 h and 504 h from their removal from the injection mold, as well as for the annealed samples. The results of DSC tests were used to determine the degree of crystallinity (*X_C_*):

(4) $$X_{C}=\frac{\Delta H}{\Delta H_{C}}\times 100\%$$

where Δ*H* is the measured enthalpy of melting and Δ*H_C_* is the melting enthalpy of the 100% crystalline PP.

### 2.6. Measurements of the Tensile Properties

The mechanical properties were determined by static elongation test using a universal testing machine Z030 Zwick/Roell (Ulm, Germany) equipped with a measuring head of a nominal load of 30 kN. The extension rate during the measurements of elastic modulus was 1 mm/min. The next stage of the measurements was carried out at a speed of 50 mm/min until the samples were broken. The tests were performed at 23 °C for 10 samples from each measurement series.

### 2.7. Measurements of Impact Strength

The impact strength of polypropylene samples was determined using the *HIT 50* pendulum impact tester by Zwick/Roell (Ulm, Germany) with a pendulum of 15 J. The samples of a rectangular shape with dimensions of 80 mm × 10 mm × 4 mm, without a notch, were used in the investigations. The impact took place on the shorter edge of the samples. The tests were performed at 23 °C for 10 samples from each measurement series.

## 3. Results

### 3.1. Shrinkage

**Figure 2:**
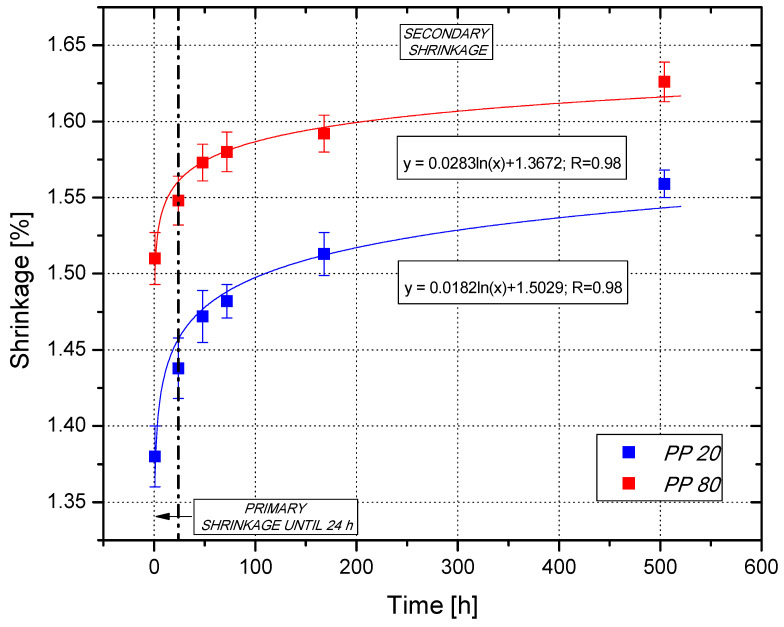
Changes of mold shrinkage values as a function of the conditioning time.

Linear processing shrinkage of the polypropylene test specimens (mold temperature of 80 °C, measurements after 1 h of conditioning) reached the value of 1.51% (Figure 2). This value was higher in comparison to the moldings produced by the mold temperature of 20 °C (1.38%), probably due to the different moldings cooling conditions. Slow cooling of a molding favors the crystallization process, which consequently results in higher shrinkage of PP80 samples. After 24 h of conditioning, the mean shrinkage value was 1.44% (PP20) and 1.55% (PP80), which corresponded to the change in the samples length of about 0.05 mm and 0.04 mm, respectively. Significant changes in the length of the moldings were recorded up to 168 h after the end of the injection process, however, even after this time, an increase in the shrinkage value of the polypropylene test specimens was noted. In the case of the samples produced by mold with *T_F_* equal to 20 °C, the shrinkage value increased from 1.51% (168 h) to 1.56% (504 h), while for the samples produced in the mold temperature of 80 °C during the same time, an increase in this value from 1.59% to 1.63% was observed. After 504 h of conditioning, the difference in value of shrinkage between the PP20 and PP80 samples was 0.07 percentage points, while after 1 h, it was 0.13 percentage points.

The changes in the shrinkage value depending on the conditioning time are logarithmic for both PP20 and PP80 samples. The trend line equations are presented in Figure 2. The obtained results confirm that the semi-crystalline polymeric products formed at higher mold temperature are characterized by a higher value of primary shrinkage, which took place in the injection mold, as well as in the first hours after sample preparation. In industrial practice, a high value of primary shrinkage of injection moldings is undesirable, however, it can be controlled by the settings of the parameters of the injection process, for example by extending the duration of the holding phase. However, the samples produced at a high temperature of the injection mold are characterized by a lower value of secondary shrinkage, i.e., a change in the dimensions of the samples, determined in this case from 24 h of conditioning. The phenomenon of secondary shrinkage is not only undesirable, but also uncontrolled, which may result in some difficulties in maintaining the dimensional accuracy of polypropylene-molded parts during their usage.

The results of shrinkage tests of PP samples annealed after 504 h of conditioning have indicated that if the conditioning process was carried out at a higher temperature or for a longer period of time, higher values of recorded shrinkage would be observed. The value of shrinkage of the annealed samples, both PP20 and PP80, was approximately 2% (Table 1). Thus, it may be stated that the annealing may reduce the difference in shrinkage values between the samples processed in different injection conditions. It is worth noting that the average length of the annealed samples was approximately 164.5 mm and was smaller by over 3 mm than the length measured at the cavity of the injection mold. The increase in the shrinkage value of PP samples after annealing results from the changes in the crystalline structure. Relatively significant changes in the shrinkage value after annealing, amounting to 0.44 (PP20) and 0.37 (PP80) percentage points, respectively, may also be the result of removing residual stresses occurring in the material after the injection process. Moreover, it can be concluded that the difference between the shrinkage values of PP20 and PP80 samples decrease along with the extension of the conditioning time.

**Figure 3:**
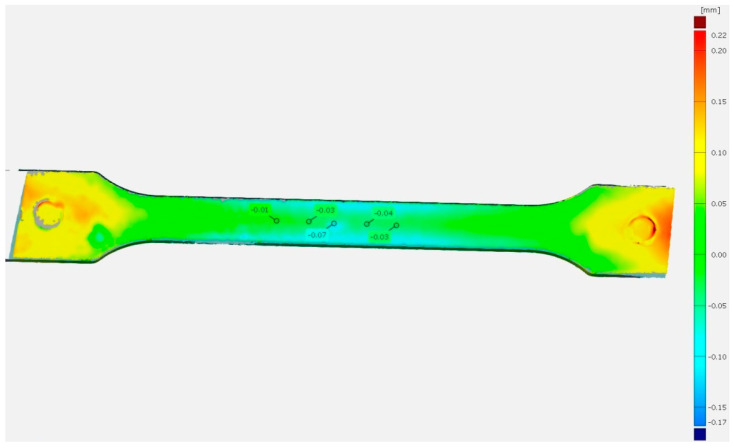
Comparison of the PP20 sample surface (reference) with the PP80 sample after 1 h of conditioning; positive deviation (red), negative deviation (blue).

The temperature value of the injection mold also influenced other dimensions of the test samples. Using a 3D optical scanner, the deviations from the reference surface were observed, particularly, the difference in the depth of sink marks on the surface of the samples (Δ*h*). The PP80 samples after 1 h of conditioning revealed a superior depth of sink marks compared to the PP20 samples after the same conditioning time. This is confirmed by the blue areas visible on the comparison of the scanned PP80 sample dot grid with the reference model (Figure 3), which was the scanned PP20 sample grid. The mean depth of sink mark in the measuring section between the two samples was about 0.04 mm.

**Figure 4:**
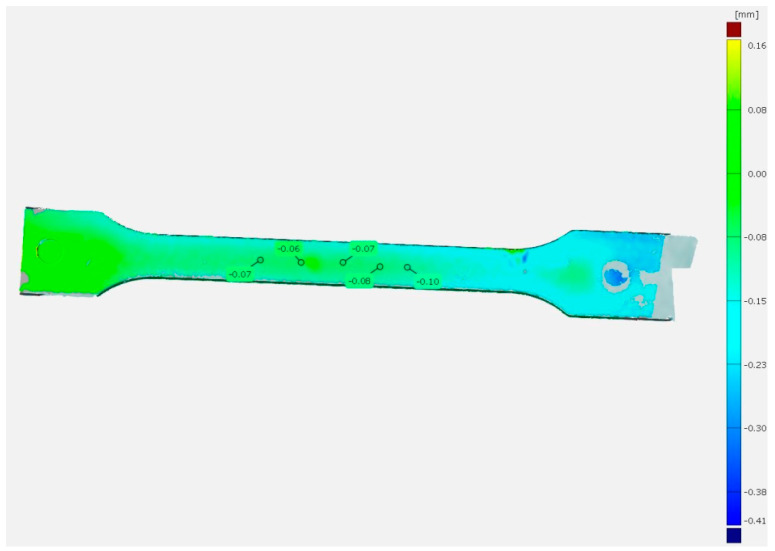
Comparison of the PP20 sample surface (reference) with the PP80 sample after 504 h of conditioning and annealing; positive deviation (red), negative deviation (blue).

Conditioning of the PP samples for 504 h, similar to the linear contraction, caused an increase in the depth of the sink marks. For the PP20 sample, the sink mark depth increased by 0.04 mm compared to the PP20-1 h sample, while for the PP80 samples, the change was 0.09 mm (Table 2). In this case, the reference sample was PP80-1 h. The consequence of heating the test samples was an additional increase in the depth of the sink marks on their surface. The values of Δ*h* in this case reached the values of 0.07 mm (PP20) and 0.014 mm (PP80). Moreover, as a result of conditioning, the difference in the depth of sink marks increased between the PP20 and PP80 samples (Figure 4). The mean value of Δh in this case was approximately 0.08 mm. The results of the study of the PP moldings shrinkage carried out with the use of 3D optical scanner indicate that the optical method can be used to evaluate even slight changes in the dimensions of injection moldings resulting from secondary shrinkage and annealing.

### 3.2. Crystalline Structure 

The changes of shrinkage values of the polypropylene samples are also accompanied by variation in the material density (Table 3). The density of the samples determined 1 h after removal of the moldings from the mold was 0.895 g/cm^3^ (PP20) and 0.899 g/cm^3^ (PP80), respectively. After 504 h of conditioning at 23 °C, the density of the samples produced in the mold at 20 °C increased to 0.902 g/cm^3^, while for the PP80 samples, it grew to 0.904 g/cm^3^, constituting an increase of approximately 0.8% and 0.5%, respectively. Although these changes are not very high, it may confirm the occurrence of the secondary crystallization during their conditioning. The degree of crystallinity determined by density (*X_ρ_*) of the PP20 samples increased by 8.1% during 504 h of conditioning. In the case of PP80 samples, an increase in *X_ρ_* by 6.1% was noted. A pronounced increase in the density was observed after the annealing of the samples, where both PP20 and PP80 samples had the same density, equal to 0.911 g/cm^3^, related to an increase in the degree of crystallinity to 70.6%. The same value of the degree of crystallinity of the annealed PP20 and PP80 samples justifies similar results of the processing shrinkage determined for the annealed samples. The increase in PP density during conditioning is the effect of the crystalline structure ordering of the material, which was also confirmed by Dudić in the studies of accelerated aging of PP samples obtained by compression molding [9]. The course of secondary crystallization was possible because PP samples were stored at a higher temperature than the glass transition temperature of this polymer.

**Figure 5:**
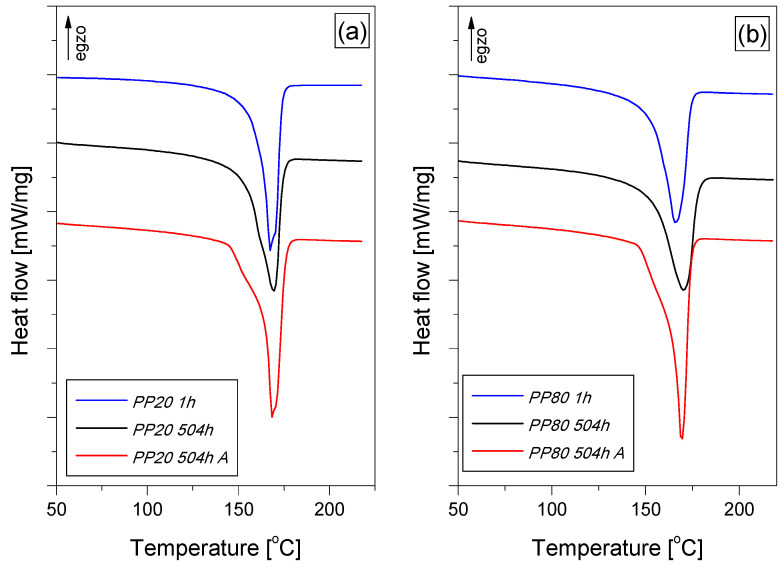
DSC melting curves of conditioned and annealed PP samples; PP20 (**a**), PP80 (**b**).

The structural changes taking place in the polymer are also confirmed by the results of calorimetric measurements. Figure 5a,b show the DSC melting curves recorded during the heating of PP samples conditioned for 1 h, 504 h and samples after annealing. The course of the DSC melting curves indicates that PP in moldings is characterized by a *α*-type crystal structure [31], with the values of the melting point in the range of 167 °C to 171 °C. Therefore, for the evaluation of the crystallinity of the samples, the value of 148 J/g was adopted as the enthalpy of melting *α*-PP with a 100% degree of crystallinity [35]. The value of melting enthalpy of PP20 samples increased from 91.39 J/g (after 1 h of conditioning) to 94.58 J/g (504 h of conditioning). The increase in the value of the melting enthalpy indicates an increase in the mass fraction of the crystalline phase in the structure. During the conditioning of the samples at a higher temperature than the glass transition temperature, the amorphous phase slowly transformed into the crystalline phase, which resulted in an increase in the degree of crystallinity by 2.2%. In the case of the PP80 samples, after the same period of time, an increase in the degree of crystallinity by 0.9% was observed. The annealing of the conditioned samples resulted in a further increase in the sample’s melting enthalpy, which corresponded to an increase in the degree of crystallinity to the values of 73.6% (PP20) and 76.2% (PP80), respectively. The observed values of melting enthalpy are slightly higher than the values determined by Dudić [8] for annealed samples that were not conditioned before. Moreover, the recorded course of the DSC melting curves of the annealed PP samples may indicate that not only quantitative changes (increase in the degree of crystallinity) but also morphological changes occurred in the crystalline structure of the material. It may be assumed that the observed total energy effect of melting of the annealed PP20 and PP80 samples consists of at least two overlapping transformations. This is indicated, inter alia, by the inflection of the melting curves at a temperature of about 163 °C.

### 3.3. Mechanical Properties

**Figure 6:**
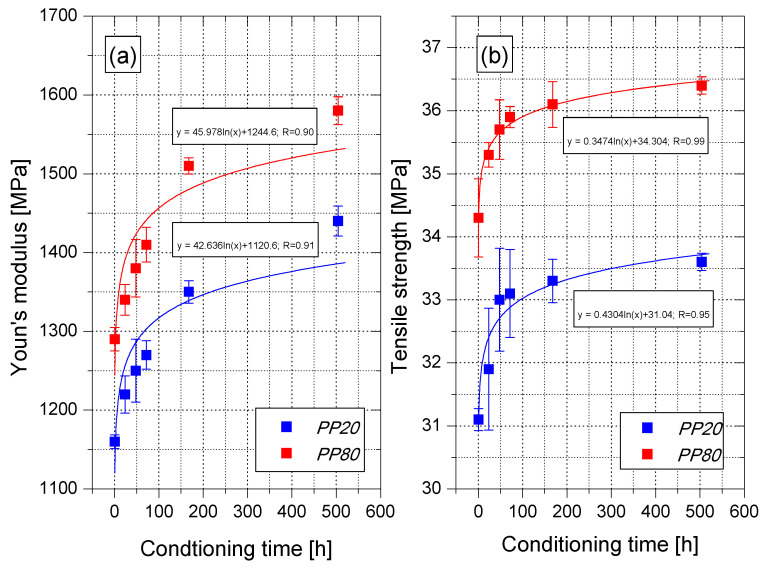
Changes of the *E* modulus (**a**) and tensile strength (**b**) of PP samples in the function of conditioning time.

The changes in the PP structure, resulting in post-molding shrinkage, influence also the changes in the mechanical properties. Slow cooling of the polypropylene in the injection mold at the temperature of 80 °C resulted in an elastic modulus value equal to 1290 MPa after 1 h of conditioning (Figure 6a). This value is about 10% higher than in the case of PP20 samples (1160 MPa). PP20 and PP80 samples tested after 24 h and 168 h were characterized by a higher value of Young’s modulus by approximately 5% and 15%, respectively, in comparison with the values determined 1 h after removing the moldings from the mold. The results of the tests, which were carried out after 504 h of conditioning, showed that the *E* value is still not stabilized. The modulus of elasticity in this case reached the values of 1440 MPa (PP20) and 1580 MPa (PP80), respectively. It should be noted that after 504 h, PP80 samples still have an *E* value higher than 10%.

**Figure 7:**
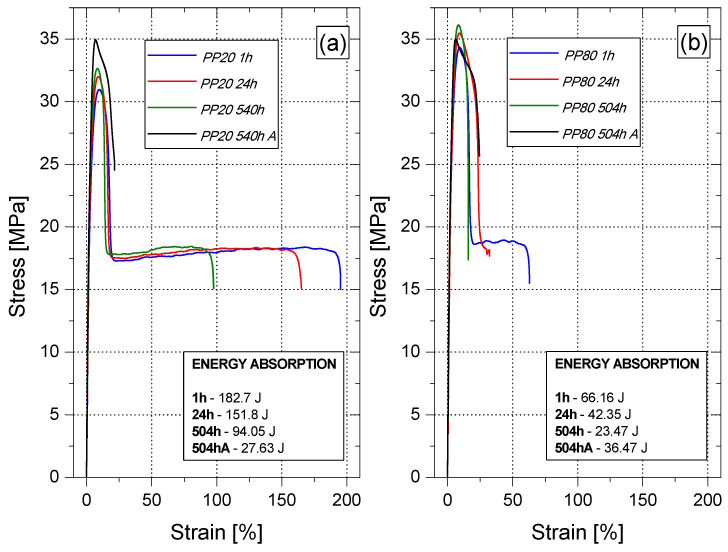
Selected stress-strain curves of conditioned and annealed PP samples; PP20 (**a**) and PP80 (**b**).

Similar trends were observed in the case of changes in the tensile strength values (6b). After 1 h of conditioning, *R_m_* of PP80 samples was equal to 34.3 MPa, being 10% higher in comparison with PP20 samples. The most significant changes in the tensile strength values were observed up to 48 h of conditioning. After 168 h of conditioning, the increase in *R_m_* value in comparison with the tensile strength after 1 h of conditioning was 7% (33.3 MPa) for PP20 and 5% (36.1 MPa) for PP80, respectively. These relative changes in values were significantly smaller than those recorded for the modulus of elasticity. However, as in the case of the modulus of elasticity, a further increase in maximum stress was recorded after 504 h of conditioning. Moreover, the observed increase in tensile strength during conditioning was significantly smaller in comparison with the values determined by Yue for PP samples obtained by compression molding [29]. The increase in the values of elasticity modulus and tensile strength is related to the increase in the content of the crystalline phase in the material structure, which is characterized by greater stiffness in comparison to the amorphous phase. Changes in the mechanical properties of PP samples during conditioning indicate the need to conduct comparative tests of injection moldings made of this material within a defined time after the end of the manufacturing process. This applies to study and development works as well as research related to quality control. The changes in the tensile strength values of PP20 and PP80 samples at individual stages of conditioning are visible in Figure 7a,b, which presents the stress–strain curves of the tested samples.

**Figure 8:**
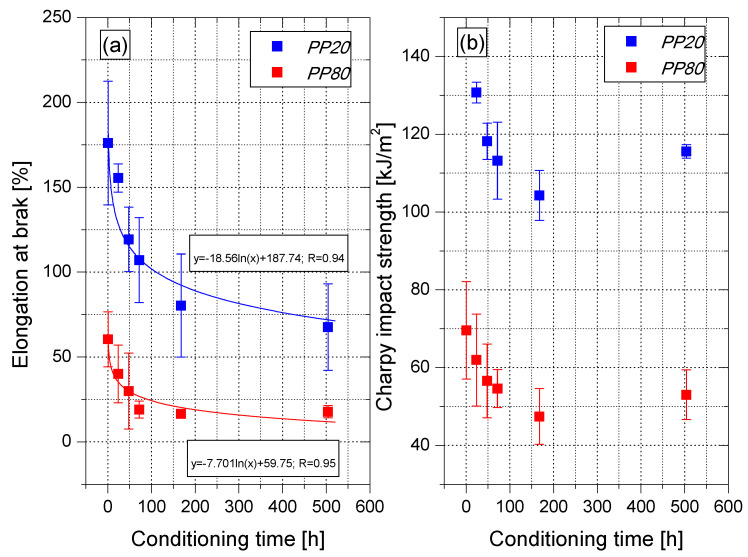
Elongation at break (**a**) and Charpy impact strength (**b**) of PP samples in the function of conditioning time.

Moreover, on the basis of the analysis of the stress–strain relationship, it can be concluded that PP80 samples are characterized by significantly lower values of elongation at break (*ε_b_*) in comparison with the PP20 samples. The value of the elongation at break of PP samples after 1 h of conditioning reached the value of 176% (PP20) and 60.5% (PP80), respectively, as shown in Figure 8a. This difference results from the lower content of the amorphous phase in the samples slowly cooled in the injection mold, which is characterized by a greater elasticity compared to the crystalline phase. As a result of the secondary crystallization taking place during the conditioning, the value of *ε_b_* gradually decreased and after 504 h from removing the moldings from the injection mold, it reached the value of 67.6% for PP20 and 16.9% for the samples injected into the mold at a temperature of 80 °C. Changes in the values of strain at break indicate an increase in brittleness of PP samples with the passage of their conditioning time. This is also confirmed by the values of the energy that were absorbed until their destruction during static tension (Figure 7a,b). For PP20 samples, the absorbed energy value decreased from approximately 183 J for the sample tested 1 h after injection to 94 J after 504 h of conditioning. The brittleness of the samples produced at the higher temperature of the injection mold was increased significantly. Immediately, after 1 h of conditioning, the registered value of absorbed energy was 66 J. After 504 h of conditioning; this value dropped to about 23 J and was comparable to the value determined for the PP20 sample after annealing.

Similar changes were observed for the Charpy impact strength up to 168 h of conditioning (Figure 8b). PP20 sample was not damaged after 1 h of conditioning, and the impact strength value after 24 h was equal to 130 kJ/m^2^. After 168 h of conditioning, the impact strength was 20% lower and reached the value of 104 kJ/m^2^; further conditioning increased the impact strength to 115 kJ/m^2^. A similar trend was observed for PP80 samples. However, the impact strength 1 h after conditioning was 69.6 kJ/m^2^. This value was significantly lower in comparison with all PP20 samples tested at different conditioning stages. After 168 h, the determined value of impact strength for PP80 was 47.4 kJ/m^2^ and increased to 53.0 kJ/m^2^ after the next 336 h. The observed increase in impact strength is probably caused by the changes in the morphology of the PP crystalline structure.

**Figure 9:**
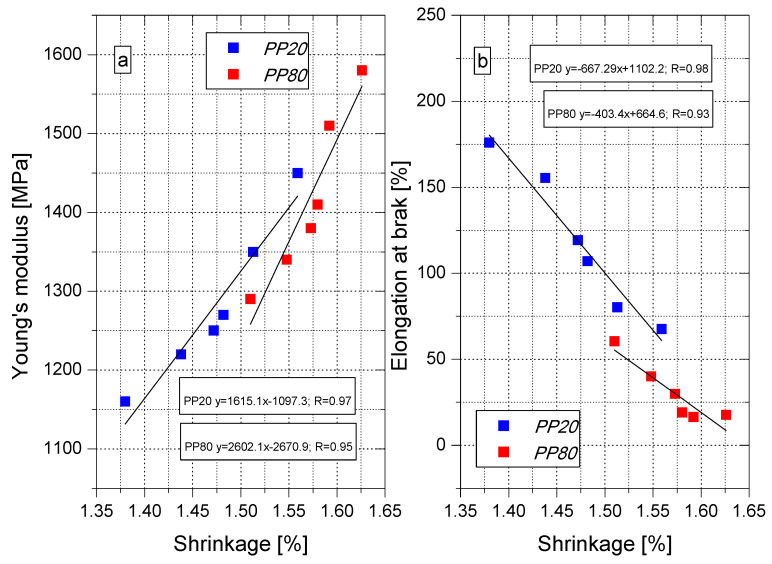
Correlation between young’s modulus (**a**), elongation (**b**) at break and shrinkage.

Based on the analysis of the test results, the relationship between the changes in the value of Young’s modulus (Figure 9a), strain at break (Figure 9b) and the changes in the shrinkage value for PP samples produced in the injection mold at 20 °C and 80 °C was determined. The value of Young’s modulus increases linearly with the increase of the shrinkage value, while the relationship between strain at break and shrinkage is inversely proportional, i.e., as the shrinkage value increases, the strain value decreases. Thus, it can be concluded that non-destructive shrinkage tests may serve as the basis for estimating the mechanical properties of polypropylene moldings.

Annealing of the conditioned PP20 and PP80 samples for 24 h at 140 °C resulted in an increase in the value of the modulus of elasticity (Table 4). Moreover, the difference between *E* values of the samples processed under different conditions decreased. The determined values were equal to 1650 MPa (PP20) and 1710 MPa (PP80), respectively. The annealed PP20 and PP80 samples were also characterized by similar values of tensile strength (35 MPa) and elongation at break (20%). However, in the case of PP20 samples, annealing resulted in an increase in the maximum stress, while for PP80, its decrease was recorded. The similar values of mechanical properties determined during the static tensile test of two series of samples are convergent with the results of shrinkage and the degree of crystallinity tests. Moreover, annealing resulted in a significant increase in the impact strength of the samples in comparison with the values recorded after 504 h of conditioning. In the case of PP20, the determined value was equal to 220 kJ/m^2^, while for PP80, it was 90.8 kJ/m^2^. The Charpy impact strength values confirm the upward tendency of this parameter value observed for the samples conditioned for 504 h. The observed increase in the impact strength of PP samples after annealing is caused by the changes in the morphology of the material’s crystalline structure, which are difficult to be clearly assessed on the basis of the conducted tests results obtained with the use of the differential scanning calorimetry method. Changes in the morphology of the crystalline structure may, for example, involve the transition of the *α*-PP phase to the *β*-PP phase, which is characterized by a higher impact strength value [13,15].

## 4. Conclusions

It has been confirmed that the post-molding time significantly affects the shrinkage and mechanical properties of the isotactic polypropylene molded parts obtained at a temperature of 20 °C and 80 °C of the injection mold. Namely, the tensile strength and Young’s modulus increased, the value of both parameters being the higher the longer the post-molding time was determined. This may be clearly observed in the case of the changes in Young’s modulus. For both types of samples, the modulus of elasticity increased by about 5% and 20% after 24 h and 504 h of conditioning, respectively, in comparison to the value determined for the samples after 1 h of conditioning. At the same time, the elongation at break and the impact strength have been reduced. The exception is the observed impact strength value increase for the samples conditioned during 504 h, as well as after annealing of all PP molded parts. Moreover, a clear difference between the impact strength values of the annealed PP20 and PP80 samples was observed. It should be emphasized that these values were higher than those recorded immediately after the samples were made. It was found that the temperature of the mold cavity, the conditioning time and the final annealing process caused significant changes in the structure of PP molded parts, which resulted in an increase in shrinkage and significant changes in mechanical properties. PP samples produced in the mold cavities at a temperature of 80 °C were characterized by a higher value of shrinkage, degree of crystallinity, Young’s modulus and tensile strength compared to PP20 samples of the same mass and thickness. Finally, it may be concluded that the processing conditions, in this case the mold temperature, as well as the post-processing ageing of the isotactic polypropylene, may significantly modify the structure and properties of the PP products, which should be considered by the processing industry. 

## 5. Future Works 

The increase in impact strength observed in these studies for the PP molded pieces conditioned for a long time, as well as its further growth after final annealing, should be an inspiration for further research. It would be advisable to consider making attempts to determine the influence of the parameters of the injection molding process, in particular the temperature of the mold cavity, on the changes in the structure of PP molded parts of various thicknesses. Further work should be focused on explaining whether morphological changes are responsible for the increase in impact strength of PP samples a long time after their manufacturing and/or after annealing. For this purpose, it would be necessary to use X-ray tests and repeat the DSC tests under very slow heating conditions. The obtained results may be very important for the production of PP molded pieces in industrial conditions, especially for the products thicker than 2.5 mm.

## Figures and Tables

**Table 1:** Shrinkage values of annealed samples.

Sample	Sample Length (mm)	Shrinkage (%)
PP20 504h A	164.54 ± 0.03	2.06 ± 0.2
PP80 504h A	164.59 ± 0.03	2.03 ± 0.02

**Table 2:** Changes in depth of sink marks in the measurement section of conditioned PP samples.

Conditioning Time (h)	Δ*h* (mm)	
	PP20	PP80
1	Reference	Reference
504	0.040 ± 0.005	0.090 ± 0.008
504A	0.070 ± 0.010	0.140 ± 0.009

**Table 3:** Physical properties of PP samples in the function of conditioning time.

Conditioning Time (h)	Mold Temperature (°C)	Density (g/cm^3^)	*X_ρ_* (%)	Melting Enthalpy (J/g)	*X_C_* (%)
1	20	0.895 ± 0.003	51.0	91.39 ± 1.13	61.7
	80	0.899 ± 0.002	56.0	95.66 ± 0.97	64.6
504	20	0.902 ± 0.003	59.1	94.58 ± 0.95	63.9
	80	0.904 ± 0.003	62.1	96.83 ± 1.25	65.5
504A	20	0.911 ± 0.002	70.6	109.0 ± 1.21	73.6
	80	0.911 ± 0.002	70.6	112.8 ± 1.04	76.2

**Table 4:** Mechanical properties of PP conditioned samples after annealing.

Mold Temperature (°C)	Young’s Modulus (MPa)	Tensile Strength (MPa)	Elongation at Break (%)	Impact Strength (kJ/m^2^)
20	1650 ± 30	34.8 ± 0.15	22.1 ± 2.6	220.1 ± 4.9
80	1710 ± 16	34.9 ± 0.25	21.4 ± 4.7	90.8 ± 17.4

## Data Availability

The data presented in this study are available on request from the corresponding author.
